# Assessment of Quality of Life among Children with End-Stage Renal Disease: A Cross-Sectional Study

**DOI:** 10.1155/2018/8565498

**Published:** 2018-09-16

**Authors:** Arwa M. El Shafei, Ibrahim Soliman Hegazy, Fatina Ibrahim Fadel, Eman M. Nagy

**Affiliations:** ^1^Department of Public Health and Community Medicine, Faculty of Medicine, Cairo University, Giza, Egypt; ^2^Department of Pediatrics Medicine, Faculty of Medicine, Cairo University, Giza, Egypt

## Abstract

**Background:**

Measuring health-related quality of life is considered an important outcome indicator in evaluating health-care interventions and treatments and in understanding the burden of diseases.

**Objectives:**

This study aimed at assessing quality of life among children with end-stage renal disease, either undergoing hemodialysis or had renal transplantation therapy and comparing it with healthy controls.

**Methods:**

A cross-sectional study was conducted between December 2016 and May 2017 in Abo El-Reesh Pediatric Hospital using parent/child reports of generic module for QoL assessment: PedsQL^TM^ Inventory version 4 for both cases and controls. Disease-specific module: PedsQL^TM^ ESRD version 3 was used for ESRD cases. 55 ESRD cases and 86 controls were enrolled in the study.

**Results:**

Statistically significant difference between ESRD cases and controls regarding all aspects of QoL was found; total QoL mean score was 58.4 ± 15.3 and 86.8 ± 10 among cases and controls, respectively. All individual QoL domains were significantly worse in ESRD cases. Transplantation group had better Spearman's correlation between child and parents' scores which showed significant positive moderate correlation.

**Conclusions:**

ESRD and its treatment modalities are affecting negatively all aspects of quality of life; incorporating QoL assessment and management is highly recommended.

## 1. Introduction

Chronic kidney disease (CKD) refers to a condition related to irreversible kidney damage that can further progress to end-stage renal disease (ESRD). CKD is a major public health problem worldwide, and extensive epidemiological research in the adult population is available. In contrast, little is known about the epidemiology of CKD in the pediatric population [1].

In Egypt, due to the absence of a national registry, the exact incidence and burden of CKD in children are not known. In a developing country such as Egypt, with limited diagnostic resources, end-stage renal disease (ESRD) is probably the “tip of the iceberg,” where patients are diagnosed with renal disease when they have already reached the end-stage renal failure [2].

Over the past decades, the scope of medical care has been changed from diagnosis and management of infectious diseases to prevention and control of chronic conditions. There was also a significant increase in reported survival rates of chronic childhood illnesses. This occurred due to advances in medical research and improvements in medical and surgical care and led to prompt to question about the relationship between the quantity and quality of survival [3, 4].

To evaluate the outcomes of medical care practices, it goes beyond clinical indicators of the disease to reach the patients' perception of their health condition and related treatment. That is why the concept of measuring quality of life (QoL) and health-related quality of life (HRQOL) has been raised [5].

So measuring HRQOL is considered an important outcome indicator in evaluating health-care interventions and treatments and in understanding the burden of diseases; it is also important in identifying health inequalities, in allocating health resources, and in epidemiological studies and health surveys. In clinical practice, it has been suggested that HRQOL instruments can be useful in identifying and prioritizing health problems for individual patients and identifying the unexpected health problems [6].

World Health Organization (WHO) defines quality of life as individuals' perception of their position in life in the context of the culture and value systems in which they live and in relation to their goals, expectations, standards, and concerns [7].

Quality of life has been investigated in several acute and chronic diseases in children such as cancer, diabetes, obesity, phenylketonuria, asthma, bipolar disorders, and scleroderma. However, measurement of quality of life gained more importance in health care as medical treatment became able to extend length of life sometimes at the expense of quality of life or improve quality of life without extending the length of life. Simple measures of death rates were no longer enough to measure changes in population health. Measurement of quality of life was also important, and there are only a few studies concerning QoL in children and adolescents with chronic kidney disease (CKD) on dialysis therapy or after renal transplantation [8].

As a result of advances in technology and medical care, children with CRD have a long-term survival. This evoked the need of measuring to what extent that improvement in survival can affect the quality of life [9].

Moreover, children with ESRD are at risk for underperforming academically, struggling socially, and experiencing adjustment difficulties and psychological stress, and children with ESRD must follow restrictive dietary and fluid regimes and a lifecycle of recurrent dialysis and transplantation to sustain life. Furthermore, dialysis treatment schedules are burdensome and interfere with school attendance and participation in peer-related activities, thereby compromising opportunities for attaining academic and psychosocial potential [9, 10].

This study aimed at investigating the quality of life among chronic renal disease children improving it through the following objectives: Assessing quality of life for children on renal dialysis and children who had renal transplantation attending Cairo University Pediatric Hospitals using a validated tool and comparing it with healthy controls then suggesting recommendations for improving quality of life for children with chronic renal disease on dialysis or had renal transplantation.

## 2. Methods

### 2.1. Study Settings

This study was conducted between December 2016 and May 2017 in Abo El-Reesh Pediatric Hospital.

### 2.2. Study Design

A cross-sectional study was performed to assess the quality of life among pediatric ESRD patients applying the PedsQL^TM^ scale, and a case-control study of the PedsQL 4.0 generic Core scale was performed to compare the HRQOL between the included ESRD children (children with end-stage renal disease who are undergoing renal dialysis (25 cases) and children who had renal transplantation (30 cases)) and 86 controls. The inclusion criteria were age 5–18 years, children with ESRD undergoing dialysis for at least 6 months, and children with transplanted kidney done since 6 months at least, while the exclusion criteria were patient had changed dialysis modalities within the past 30 days, patient had been hospitalized within the last 14 days (with exclusion of hospital stay for dialysis), and the child had experienced a significant life event unrelated to their kidney disease in the past 30 days, such as the death of a family member.

### 2.3. Sampling

All children satisfying the inclusion criteria were enrolled in the study; they constituted 25 cases out of 46 dialysis patients, while the transplantation patients were 30 cases (fulfilling the inclusion criteria) out of 100 patients as almost 60% of patients were within older age group. Controls were age- and sex-matched, and they were 86.

### 2.4. Data Collection Tools

A-*Arabic PedsQL*^*TM*^*Inventory version* 4 was used for both cases and controls: This form includes parent and child reports. It includes four domains covered by 23 questions: physical domain, emotional domain, social domain, and school domain. Both the child and the parents were asked about how much of each problem has been presented during the past one month, giving a score 0 if it is never a problem, 1 if it is almost never a problem, 2 if it is sometimes a problem, 3 if it is often a problem, or 4 if it is almost always a problem. *B- PedsQL*^*TM*^ End-Stage Renal Disease Module was used for only cases. This form includes parent and child reports. It includes seven domains covered by 23 questions: general fatigue, kidney disease-related problems, treatment-related problems, family and beer interaction, worry, perceived physical appearance, and communication. Both the child and the parents were asked about how much of each problem has been presented during the past one month, giving a score 0 if it is never a problem, 1 if it is almost never a problem, 2 if it is sometimes a problem, 3 if it is often a problem, or 4 if it is almost always a problem.

### 2.5. Scoring System for Assessing Quality of Life

5-point Likert scale from 0 (never) to 4 (almost always).

Scoring Procedure. *Step 1*: transform Score items are reversed scored and linearly transformed to a 0–100 scale as follows: 0 = 100, 1 = 75, 2 = 50, 3 = 25, 4 = 0.

(So 100 score means best quality, while 0 score means worst quality of life). *Step 2*: calculate Scores Score by Dimensions: If more than 50% of the items in the scale are missing, the scale scores were not computed. *Mean score* = sum of the items over the number of items answered.

### 2.6. Data Management and Statistical Analysis

Data were coded and entered into Microsoft excel sheet and then transferred to SPSS program version 23. Descriptive statistics were presented as percentage, arithmetic mean, and standard deviation. Statistical tests of significance were used to compare between studied cases and controls where *P* value < 0.005 is considered statistically significant. Chi-square test was performed for qualitative data such as sex and education level, while Student's *t*-test or ANOVA test was performed for the quantitative data such as QoL mean scores between different groups. Spearman's correlation was performed between child and parents' QoL mean scores, where correlation coefficient ±0.9 to 1 indicates very high positive/negative correlation, ±0.7 to 0.9 indicates high positive/negative correlation, ±0.5 to 0.7 indicate moderate positive/negative correlation, ±03 to 0.5 indicates low positive/negative correlation, and 0.00 to ±0.3 indicates negligible correlation [11], while the correlation is considered significant at 0.01 level. Interrater reliability between parent and child was assessed with the intraclass correlation coefficient (ICC). The ICC is used to determine the agreement between two different raters of the same.

## 3. Results

The sociodemographic data of the included ESRD cases and controls are summarized in Table 1. The mean age of the ESRD cases was 11.9 ± 3.1, while the mean age of controls was 7.9 ± 2.7 years. It was found that about 50% of dialysis patients versus only 6% of transplantation patients were not enrolled in school.

The child report mean and parent report scores of quality of life domains (physical, emotional, social, and school domains) are summarized in Table 2. It shows that controls gave a higher mean score for all domains rather than ESRD cases. It shows also that the total QoL mean score was 58.4 ± 15.3 SD for ESRD cases, while that of controls was 86.8 ± 10 SD. Both individual domains and total score gave statistically significant difference. The parent of controls gave a higher mean score for all domains rather than ESRD cases (82.4 ± 10 and 58 ± 16.7, respectively).

The child report mean scores of quality of life domains among the ESRD subgroups (dialysis and renal transplantation) are summarized in Table 3. This shows that the mean scores for physical and school domains are higher within the transplantation group rather than the dialysis group. This difference is statistically significant (*P* value < 0.05). However, the mean scores for emotional and social domains are higher within dialysis subgroup rather than transplantation subgroup. This difference is statistically not significant (*P* value > 0.05). The total QoL mean score is 60.4 ± 16.6 and 55.9 ± 13.5 in both transplantation and dialysis subgroups, respectively. This difference is statistically not significant (*P* value > 0.05).

Regarding agreement between child and parent mean scores, Table 4 shows that there was a moderate to good agreement between child and parent reports among studied groups for both quality of life domains and total generic scale (ICC ranged from 0.61 to 0.80).

The child report mean scores of PedsQL^TM^ End-Stage Renal Disease Module domains are summarized in Table 5. There is a statistically significant difference between dialysis and transplantation groups regarding renal disease-related problems and treatment-related problems (*P* value < 0.05). It shows that there is significant difference between dialysis and transplantation groups regarding renal disease-related problems mean score and treatment-related problems mean score (*P* value < 0.05). As well as, there is statistically significant difference in the total mean score (*P* value < 0.05).

**Figure 1:**
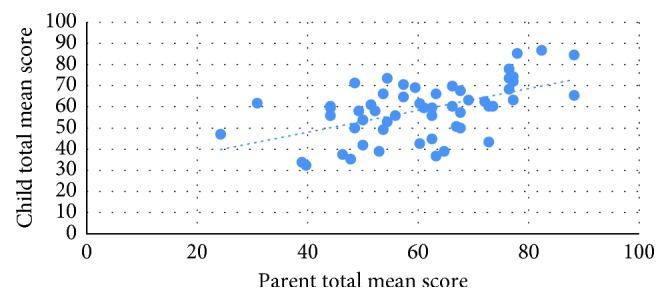
Correlation between total mean scores of ESRD QoL among ESRD cases and their parents.

Correlation between child and parent mean scores of ESRD module is displayed in Figure 1 which showed significant positive moderate correlation between child report total ESRD QoL mean score and parent report total ESRD QoL mean score among ESRD cases (*r*=0.538) (*P* ≤ 0.001).

Testing the degree of agreement between the child's mean score and that of his parent's showed excellent degree of agreement among the transplantation group (intraclass correlation ranged from 0.81 to 1.00, i.e., 0.86 confidence interval 0.67–0.93). In contrast, it shows there was a poor agreement among the dialysis group (ICC is less than 0.40, i.e., 0.28 confidence interval 0.63–0.68).

In studying the relation between the child report QoL mean scores and the current educational status of the child, there is significance difference for the physical mean score between children enrolled in school and children who are not enrolled in school (*P* value < 0.05), while there is no statistically significant difference for other domains as well as total score.

## 4. Discussion

In this study, it was found that the quality of life assessed by Generic PedsQL^TM^ is significantly lower in children with ESRD than corresponding healthy children. This was seen in both child and parent reports. Although there is no statistical difference between dialysis and transplantation subgroups, it was still obvious that in the dialysis subgroup, the total score was the lowest. This result is supported by a study conducted by Goldstein et al. and Kul et al., who concluded that ESRD especially the dialysis group gave lower scores than healthy controls [12, 13].

Lower scores for ESRD patients could be explained by clinical facts that ESRD patients suffer from many short-term and long-term complications that alter their life such as frequent hospitalization, painful medical procedures, school absence, and restriction of activities which have behavioral and negative emotional outcomes [14].

Regarding the physical functioning score, this study found that both dialysis and transplantation groups had lower scores than healthy controls. Furthermore, dialysis subgroup had significantly worst score. This finding is supported by other studies using the same tool (Generic PedsQL^TM^) as McKenna et al., Goldstein et al., and Kul et al. [12, 13, 15]. This finding may be attributed to a very common CKD complication known as chronic kidney disease-mineral and bone disorder (CKD-MBD). CKD-MBD is defined as a systemic disorder of mineral and bone metabolism due to CKD that is manifested by abnormalities of calcium, phosphorus, parathyroid hormone (PTH), or vitamin D metabolism, abnormalities in bone histology, linear growth, or strength, and vascular or other soft tissue calcification [16].

Regarding emotional and social functioning, this study found that both dialysis and transplantation subgroups had significantly lower score than controls. This finding is supported by many other studies that found also that emotional and social well-being is less in dialysis and transplantation groups than in healthy controls [8, 12, 16–18].

In general, the lower psychological values among children receiving dialysis may reflect their sense of lack of control, social isolation, poor self-esteem, perceived inability to gain independence, uncertainties about their health and future, and lifestyle restrictions attributable to the time-consuming and ongoing dialysis regimen [19]. In addition for transplantation patients receiving immunosuppression therapy, they may experience appearance changes including the Cushingoid facies and growth retardation related to long-term daily corticosteroid administration and the hirsutism and gingival hypertrophy [14].

In contrast with physical score, the dialysis group had a better score than the transplantation group for emotional and social functioning scores. This result is supported by the study of McKenna et al. who explained this by “response shift” theory which indicates that patients with chronic illness (as dialysis recipient) make internal defense mechanisms to overcome negative feelings as living with their illness becomes a routine [15]. Moreover, the school functioning QoL score is lower in ESRD cases rather than controls, with better scores for the transplantation group. This result is supported by other studies conducted by Buyan et al. in 2010, Goldstein et al. in 2006, and Kiliś-Pstrusińska et al. in 2013 [8, 11, 20].

In this study, although parents gave a lower score for all Generic QoL dimensions than children themselves, there was a significant moderate positive correlation between the two scores as well as a moderate agreement between them for all studied groups (controls, transplantation, and dialysis). A similar result found by Goldstein et al. in 2008 with a mild to moderate agreement between child and parent scores [17]. As well as, other study done by Kilis'-Pstrusinska et al. explained the better scores given by children with the possibility of their positive and temporal orientation of the life situation. As well as, parents are always thinking of the future difficulties and uncertainties [21]. This explanation is supported with the conclusion reached by Goldstein et al. and Buyan et al. about improvement of child and parent scores agreement as the child get older [8, 17].

Additionally, this study assessed the quality of life among ESRD cases (dialysis and transplantation) using a disease-specific questionnaire: PedsQL^TM^ End-Stage Renal Disease Module. It was found that transplantation patients' scores are significantly better than dialysis patients' score regarding renal disease- and treatment-related problems. This result confirms that successful renal transplantation remains the primary goal of programs that care for children with ESRD [14].

Regarding parent correlation and agreement with child scores of ESRD module, it showed a moderate positive correlation between child and parent reports with excellent agreement within the transplantation group and a poor agreement within the dialysis group. This goes with Goldstein et al.'s study that showed a poor to moderate agreement between child and parent reports of ESRD module domains [17].

*Strengths and limitations of this study.* Investigating both the child and his parent using a validated Arabic PedsQL^TM^ Inventory version 4 is considered a strength of this study. Having matched controls is another point of strength. However, this study has some methodological limitations; as completion of QoL questionnaire was done in-center dialysis unit for dialysis patients, so the surrounding environments of dialysis may have influenced the child or parents' responses.

## 5. Conclusion and Recommendations

It is quite evident in this study that ESRD affects negatively all life dimensions including physical, social, emotional, and school functioning as well. And this raises the need to introduce quality of life assessment as a part of pediatric ESRD management. As well as, careful monitoring, adjusting life obstacles, or immunosuppression therapy adjustment may have a role to improve QoL more among this group.

Quality of life assessment is a primary subjective matter. So whenever the person of concern is available, it is better to be asked. But in certain situations, if the child was too young to answer, mentally affected, or clinically cannot give answers, parents would be a good option for obtaining a child's quality of life prospection as there is a moderate to good agreement between child and parent scores; as well as, there is a positive moderate correlation between them.

It is recommended that

- Management of pediatric ESRD must include QoL assessment and management.
- The Generic PedsQL Inventory version 4 is a good tool for quality of life assessment among Egyptian children.
- PedsQL End-Stage Renal Disease-specific module is appropriate to use among children with end-stage renal diseases for assessing the impact of disease condition on their quality of life.
- Although there is a correlation between parent-proxy and child report scores for assessing the quality of life, it is better to ask both parent and child himself to get a comprehensive image of quality of life.
  - Special school tutorial programs must be developed for children with chronic kidney disease to improve school achievement.
  - Further studies on national or multicentric level should be conducted to develop a special program for quality of life improvement.

## Figures and Tables

**Table 1:** Sociodemographic data of ESRD cases and controls.

Sociodemographic characteristic	ESRD cases (*n*=55)		Controls (*n*=86)		*P* value
	*N*	%	*N*	%	
*Sex*					
Female	27	49.1	34	39.5	0.173
Male	28	50.9	52	60.5	
*Residency*					
Urban	29	52.7	63	73.3	0.01
Rural	26	47.3	23	26.7	
*Consanguinity*					
Positive consanguinity	31	56.4	23	26.7	0.001
Negative consanguinity	24	43.6	63	73.3	
*Age categories*					
5----	11	20	63	73.3	<0.001
10----	34	61.8	20	23.3	
15----	10	18.2	3	3.5	
*Current educational status*					
Enrolled in school	41	74.5	85	98.8	<0.001
Not enrolled in school	14	25.5	1	1.2	

**Table 2:** Child and parent mean scores of quality of life domains.

Quality of life domains	Quality of life domains (child report) mean ± SD	Quality of life domains (parent report) mean ± SD
*Physical QoL mean score*		
Case	58.5 ± 18.5	51.7 ± 23.4
Control	84.7 ± 16.6	79.4 ± 17.2
*Emotional QoL mean score*		
Case	64.2 ± 24.5	65.3 ± 25
Control	85.9 ± 17.1	79.7 ± 18.4
*Social QoL mean score*		
Case	61.6 ± 29.4	72 ± 25.1
Control	94 ± 9.1	93.5 ± 8.6
*School QoL mean score* ^*∗*^		
Case	46.1 ± 24.3	43.2 ± 25.9
Control	83.7 ± 14.7	78.3 ± 15.9
*Total QoL mean score*		
Case	58.4 ± 15.3	58 ± 16.7
Control	86.8 ± 10.1	82.4 ± 10.3
*Pvalue*	<0.001	<0.001

^*∗*^Assessed only for children who are enrolled in school.

**Table 3:** The mean scores of quality of life domains among the ESRD subgroups (dialysis and renal transplantation).

Quality of life domains (child report)	*N*	Mean ± SD	*P* value
*Child physical QoL mean score*			
Transplantation	30	64.5 ± 17.7	0.004
Dialysis	25	51.2 ± 16.8	
*Child emotion QoL mean score*			
Transplantation	30	62.3 ± 25.5	0.44
Dialysis	25	66.6 ± 23.4	
*Child social QoL mean score*			
Transplantation	30	58.3 ± 32.2	0.173
Dialysis	25	65.6 ± 25.6	
*Child school QoL mean score*			
Transplantation	28^*∗*^	53.1 ± 23.2	<0.001
Dialysis	13^*∗*^	31.1 ± 20.1	
*Child total QoL mean score*			
Transplantation	30	60.4 ± 16.6	0.179
Dialysis	25	55.9 ± 13.5	

**Table 4:** Agreement between child and parent mean scores of quality of life domains and total generic scale.

Groups	Intraclass correlation (ICC)	Confidence interval	*P* value
*Total cases and control*			
Total generic scale	0.869	0.817- 0.906	<0.001
*Transplantation group*			
Total generic scale	0.749	0.473–0.881	<0.001
Physical scale	0.741	0.455–0.877	<0.001
Emotional scale	0.571	0.099–0.796	0.013
Social scale	0.731	0.435–0.872	<0.001
School scale	0.742	0.442–0.881	<0.001
*Dialysis group*			
Total generic scale	0.719	0.362–0.876	0.001
Physical scale	0.58	0.47–0.815	0.19
Emotional scale	0.642	0.188–0.842	0.007
Social scale	0.245	−0.714–0.667	0.249
School scale	0.807	0.368–0.941	0.004
*Control group*			
Total generic scale	0.635	0.439–0.762	<0.001

**Table 5:** The child and parent mean scores of PedsQL^TM^ End-Stage Renal Disease Module.

	Child report mean ± SD	Parent report mean ± SD
*General fatigue mean score*		
Transplantation	67.1 ± 29.1	62.71 ± 27.83
Dialysis	55 ± 19.8	54.75 ± 26.59
*Renal disease problems mean score*		
Transplantation	79.3 ± 15.6^*∗*^	80.37 ± 18.19^*∗*^
Dialysis	58.8 ± 16.2^*∗*^	53.4 ± 14.84^*∗*^
*Treatment problems mean score*		
Transplantation	80.4 ± 22.9^*∗*^	76.46 ± 26.35^*∗*^
Dialysis	58.7 ± 23.7^*∗*^	48.5 ± 26.96^*∗*^
*Family and beer interaction mean score*		
Transplantation	58.3 ± 21.5	61.67 ± 30.61
Dialysis	51 ± 25.7	61.67 ± 22.69
*Worry mean score*		
Transplantation	61.2 ± 17.8	61.08 ± 14.75
Dialysis	59.3 ± 19	53 ± 16.11
*Perceived physical appearance mean score*		
Transplantation	49.2 ± 35.5	47.43 ± 34.68
Dialysis	54.3 ± 30.6	56.67 ± 26.46
*Communication mean score*		
Transplantation	48.3 ± 26.5	49.17 ± 29.25
Dialysis	55.2 ± 23.3	49.2 ± 27.41
*ESRD module total mean score*		
Transplantation	63.6 ± 13.6	63.01 ± 12.69^*∗*^
Dialysis	56.8 ± 13.3	53.26 ± 12.04^*∗*^

## Data Availability

Data of both the Generic and ESRD PedsQL used to support the findings of this study are available from the corresponding author upon request.
